# The Air Inside Joint: A Sign of Disease Pathology or a Benign Condition?

**DOI:** 10.7759/cureus.8479

**Published:** 2020-06-06

**Authors:** Krishnan Nagarajan, Pravash Mishra, Sandeep Velagada, Sujit K Tripathy

**Affiliations:** 1 Radiodiagnosis, Jawaharlal Institute of Postgraduate Medical Education and Research, Puducherry, IND; 2 Anatomy, All India Institute of Medical Sciences, Bhubaneswar, IND; 3 Orthopaedics, All India Institute of Medical Sciences, Bhubaneswar, IND

**Keywords:** pneumoarthrosis, vacuum phenomenon, intra-articular air, radiographs, radiolucent shadow

## Abstract

‘Vacuum phenomenon’ or ‘pneumoarthrosis’ term is used when there is air within a joint space. It has been described commonly in the spine and occasionally in the peripheral articulations. It is usually seen following trauma, and sometimes spontaneously in joints showing degenerative changes. Although it has been mainly described with a conventional radiograph, other diagnostic modalities such as ultrasonography, CT scan, and MRI have also been used for its delineation. We present three cases of vacuum phenomenon observed in the shoulder joints and the hip joint. These radiolucent shadows were visible in the radiograph and vanished subsequently. The ‘air inside the joint’ may be a benign condition and does not necessitate further workup unless the patient is symptomatic.

## Introduction

Pneumoarthrosis, i.e. air within a joint cavity, can be seen in the radiograph of a joint. This air entry could be due to the development of negative pressure within the joint cavity [1,2]. Because of sudden contraction of the surrounding muscles, there is a distraction of the joint, resulting in an increase in the joint volume creating a negative pressure or the development of pressure gradient across the joint cavity. In disc spaces, it may occur due to a degenerative phenomenon. Gases like nitrogen, dissolved in the surrounding tissue, are at slightly higher pressure. Because of the development of pressure gradient across the joint cavity, these gases rapidly diffuse into the joint cavity and outline the synovial layer or the articular cartilage not covered by the synovium, and form a curvilinear lucency in the radiograph. It is commonly seen in large joint cavities like shoulder and hip, but can be seen in smaller joints too [1,2].

The presence of gas within a joint space was first described by Fick in 1910, and subsequently, it was reported in the intervertebral disc, pubic symphysis, peripheral synovial joints with open injury, with the traction of the joints as in hip, with infective spinal causes, non-open injury as in ankle and temporomandibular joint (TMJ), and mostly with degenerative changes in all the joints [2-8]. Although routine radiographs have been mainly used to demonstrate vacuum phenomenon, other modalities such as ultrasonography (USG), CT scan (CT), and MRI can better delineate the air shadow. CT is pathognomonic as the vacuum phenomenon is easily identifiable due to low attenuation values of gas [6,8,9]. Initially, it was thought that the presence of air excludes the presence of joint effusion; hence, serious injury was excluded, but few reports had shown the presence of joint effusion when radiographs showed lucency of pneumoarthrosis [10,11]. So simple presence of vacuum phenomenon does not exclude other serious pathology, and further workup should be done. We present three cases of pneumoarthrosis in the shoulder and hip joints and discuss the available literature on intra-articular air.

## Case presentation

**Figure 1:**
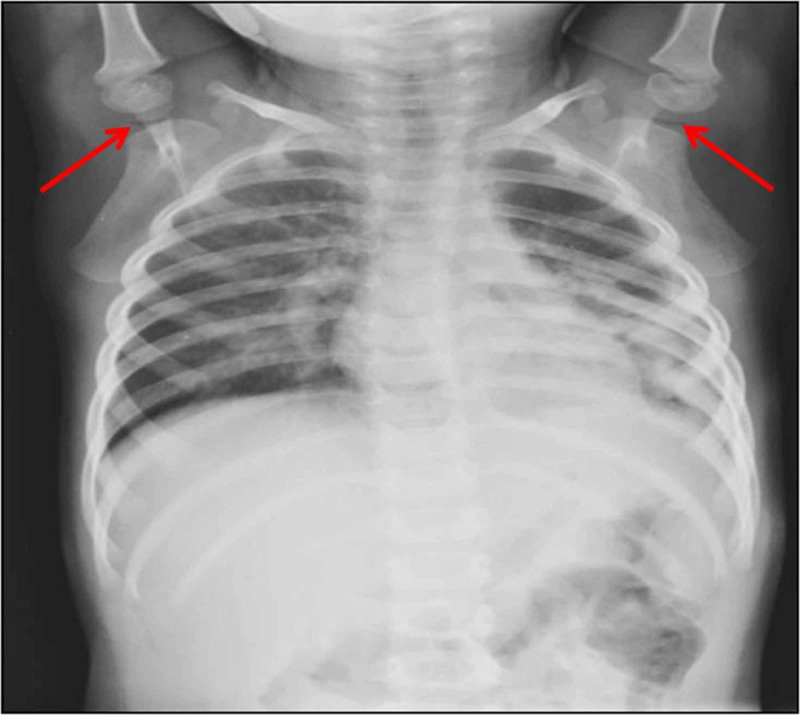
Chest radiograph of a three-year infant showing pneumoarthrosis in shoulder joints

**Figure 2:**
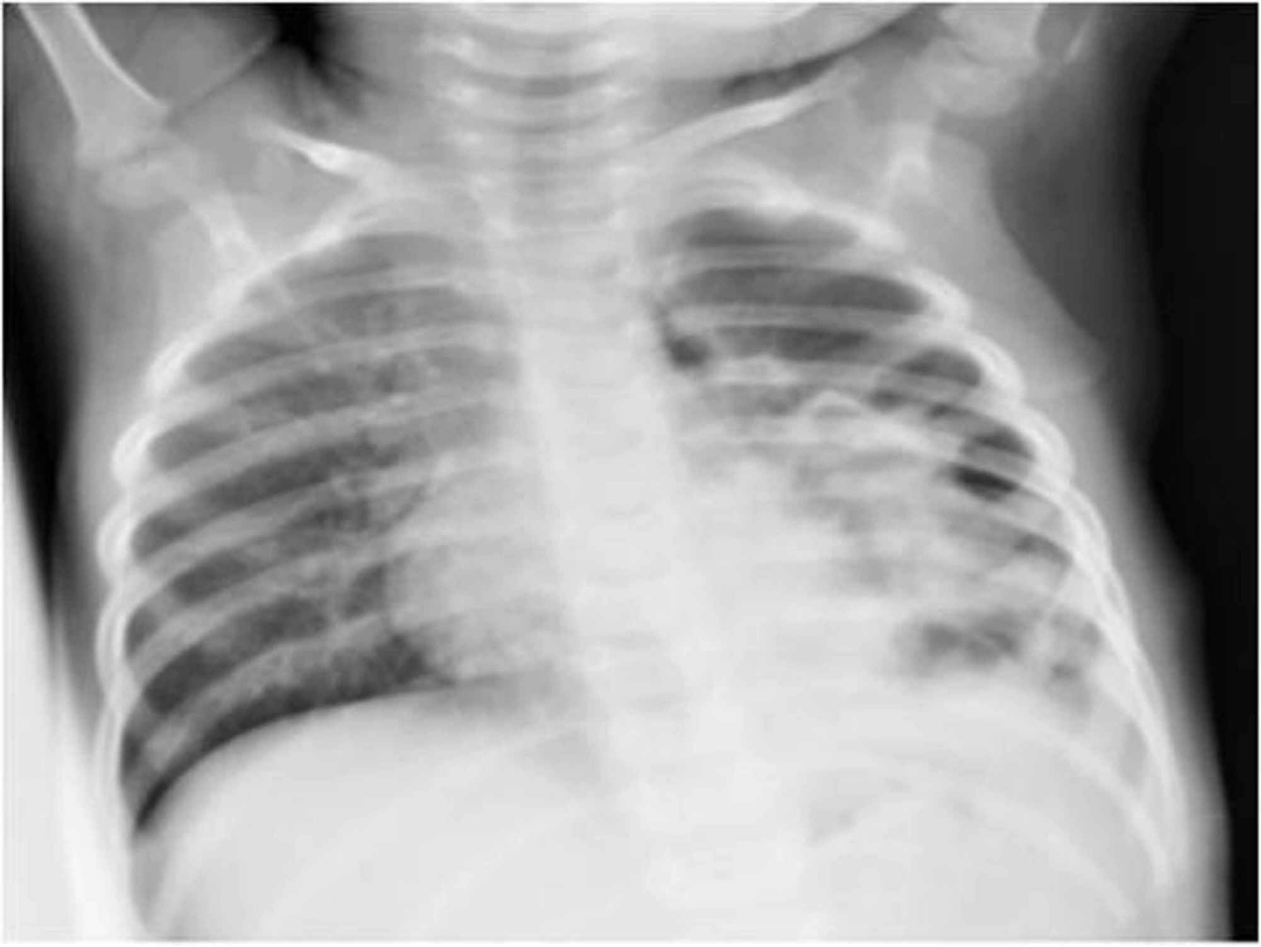
Follow-up radiograph shows the absence of intra-articular air

A three-year-old male child was brought for a chest radiograph due to respiratory symptoms. Apart from left perihilar opacities, bilateral glenohumeral joints showed lucencies due to the vacuum phenomenon with arms of the infant kept above the head. In this case, it might have resulted from overhead abduction of the shoulder joint resulting in joint distraction. The follow-up chest radiograph did not reveal any air in the shoulder joints. However, the left perihilar opacities were still seen in the follow-up x-ray (Figures 1, 2). It indicated that the vacuum phenomenon was not pathological.

**Figure 3:**
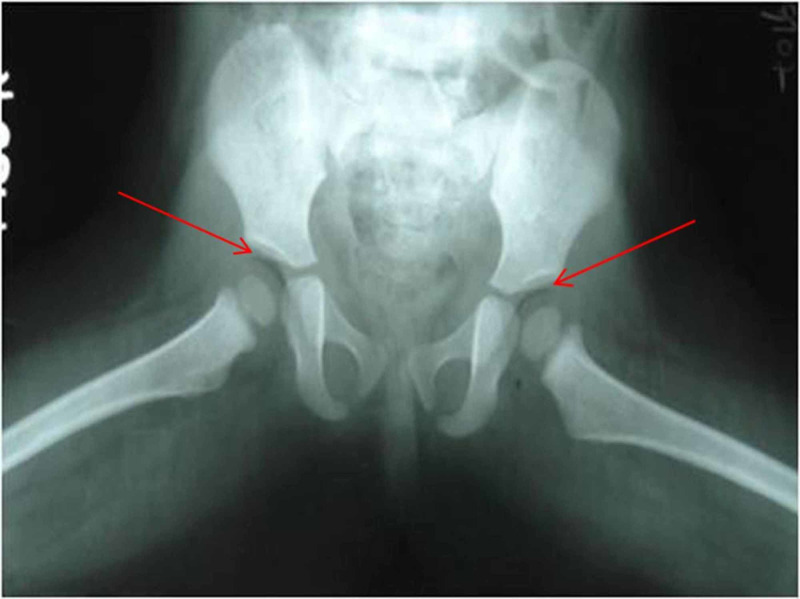
Frog-leg position radiograph of hip joints in an eight-month infant showing pneumoarthrosis

**Figure 4:**
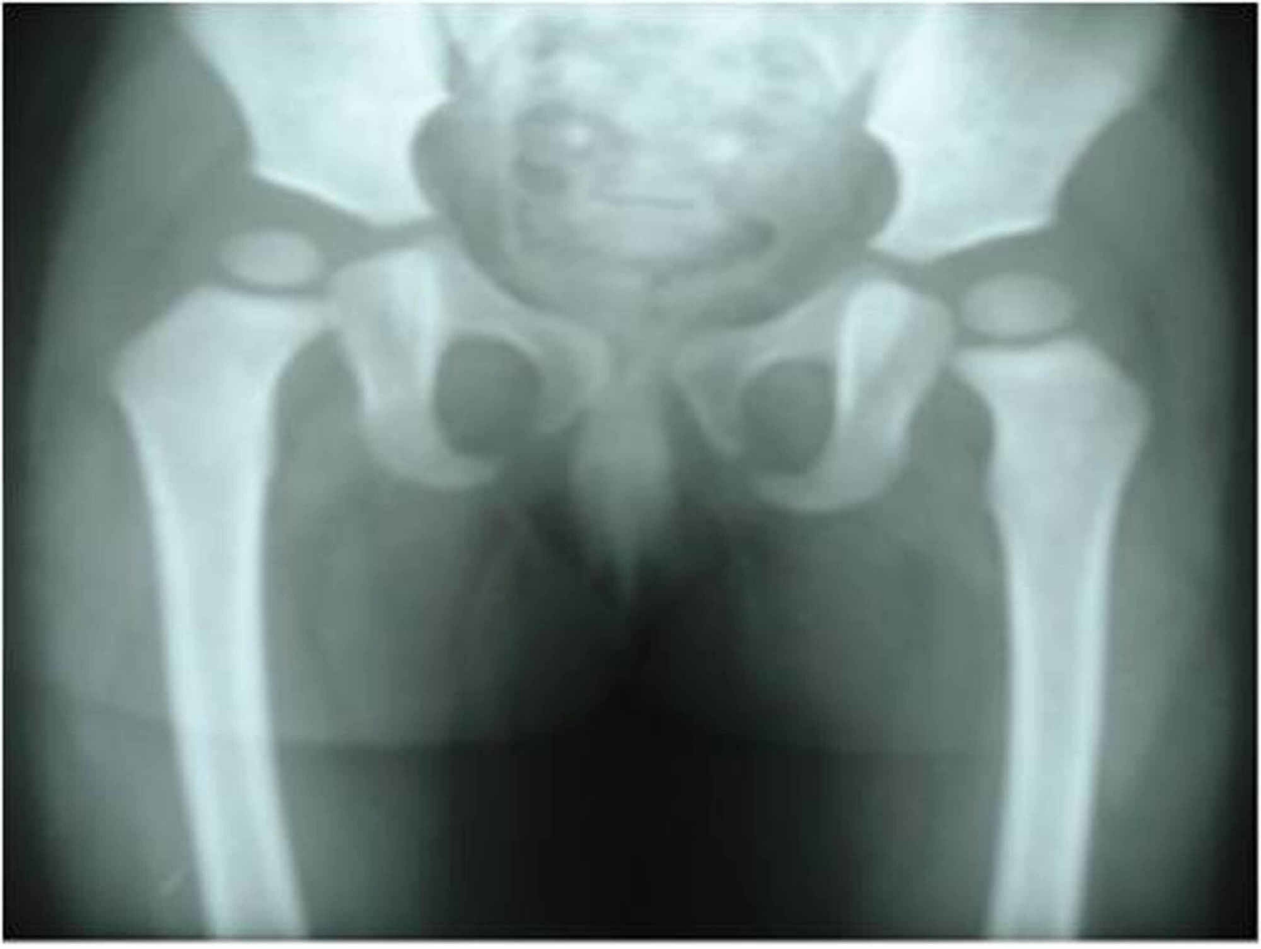
Disappearance of gas shadow in follow-up anteroposterior (AP) view

In the second example, intra-articular air was noted in the frog-leg lateral view of hip joints (in abduction) in an eight-month-old infant suspected of hip dysplasia, but follow-up radiograph did not show the vacuum phenomenon (Figures 3, 4).

**Figure 5:**
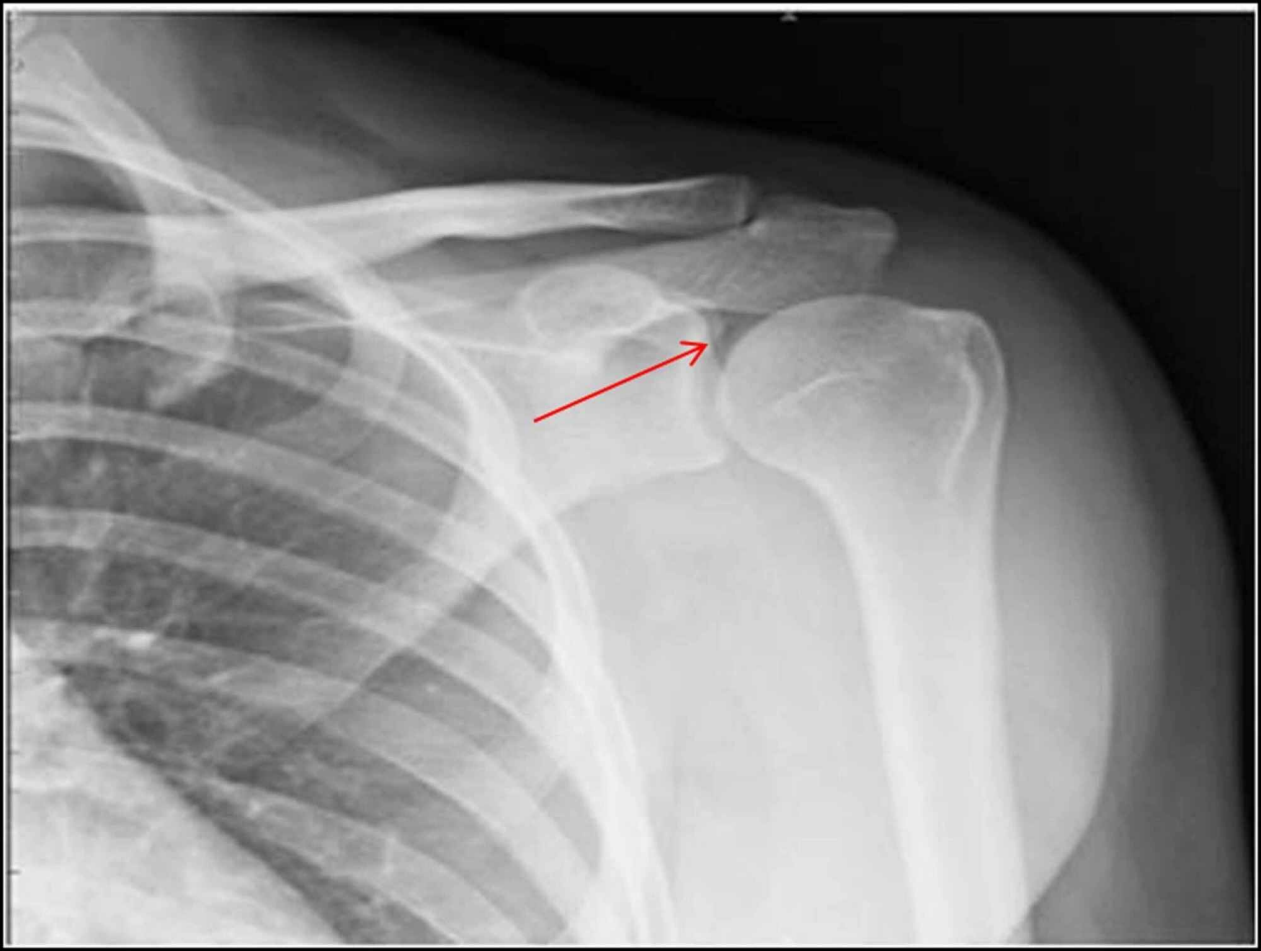
Left glenohumeral joint in 28-year-old male showing thin crescent of intra-articular air with mild external rotation of arm (reduced overlap of glenoid labrum and humeral head)

The third radiograph in an adult showed a thin crescent of air in the left glenohumeral joint probably due to a slight external rotation of the arm, as seen with the reduced overlap of glenoid rim and humeral head. In all these three instances, follow-up clinical and radiological examination showed clinically normal joints (Figure 5).

## Discussion

Pneumoarthrosis or vacuum phenomenon can be broadly classified by location into spinal and non-spinal categories. History of trauma should be asked when the vacuum phenomenon is observed in non-spinal locations. It is more common in open/penetrating type injury followed by the closed type. It can be spontaneous (without traction) and sometimes, it can be seen following traction of the joint. The presence of traction with clear delineation of pneumoarthrosis has been reported in the spine with extension, in the hip joint with the abduction and external rotation, in the sternoclavicular joints with elevated arms, in the pubic symphysis during pregnancy, in the glenohumeral joint with external rotation, and in the TMJ with wide-open mouth position [2].

Pneumoarthrosis may be seen in infants, particularly around the shoulder joint when the arms are suddenly and fully abducted while positioning for chest radiograph in frontal projection. It can be visualized in the hip joints following sudden abduction of the femur, though not commonly seen in all radiographs. The time interval between positioning the patient and taking the radiograph should be less to illustrate the pneumoarthrosis. The increase in the time interval between positioning and imaging may be one of the factors responsible for the absence of this sign in many radiographs. Ray et al. reported a case of a vacuum phenomenon in the shoulder of a child following a road traffic accident without any fracture or dislocation. They stated that in the absence of any fracture, vacuum phenomenon could be an indicator of underlying soft tissue injury [12]

After initial reports of the presence of intra-articular air, many studies showed that traction may cause the vacuum phenomenon in the joint [2,13]. Arvidsson studied the force necessary to create a vacuum phenomenon and reported that a force of 400 N was necessary to achieve traction on the hip joints, and a force of 400-600 N for pneumoarthrosis [13]. The contents of pneumoarthrosis were analyzed by Ford et al., who used a closed discography system to aspirate the air in the intervertebral disc [14]. With gas chromatography, they reported 90%-92% of contents were due to nitrogen.

The degenerative joint disease produces a vacuum phenomenon in sacroiliac joints, ankles (tibiotalar and subtalar), and in the craniovertebral junction (CVJ, C1-2). Although the vacuum phenomenon has been described in sternoclavicular and sacroiliac joints, there was no difference between traumatic and non-traumatic groups [2]. The correlation of the vacuum phenomenon in sacroiliac joints and sacropelvic morphology has been studied. It was more common in females compared to males. Although the presence of a vacuum phenomenon did not affect the lumbopelvic parameters in females, there was a significant decrease in pelvic incidence and sacral slope in males with a vacuum phenomenon [15]. A study by You et al. concluded that prevalence of vacuum phenomenon in sacroiliac joint in children is the same as that of adults; however, they were not able to determine the association between degenerative changes in the sacroiliac joint and low back pain among children and adolescents [16]

In acute open injury, the presence of air indicates communication of joint space to outside, even as an iatrogenic cause as in arthroscopy complicated by tension pneumoarthrosis [2]. The presence of intra-articular air may simulate meniscal and cartilaginous injury in the knee, but Miller et al. and Wright et al. showed the presence of air was actually associated with meniscal and bony injuries, respectively, in their patients`[12,17]. Few authors reported air in ankle and TMJ. They revealed that an intact capsule is a requisite for pneumoarthrosis even though there may be adjacent bony injuries like subtalar fracture dislocation in the ankle joint and condylar fracture in the TMJ [15]. The vacuum phenomenon is most commonly seen in intervertebral discs. Murata et al. from their study concluded that the vacuum phenomenon in intervertebral disc is associated with lumbar disc degeneration and canal stenosis as evaluated by MRI [18].

Vertebral pneumatocysts have been followed up with MRI, which showed fluid replacement of the air cyst. There may be two courses of progression and regression of vertebral pneumatocysts, usually in younger and elderly, respectively [2]. Although a degenerative vacuum phenomenon has been noted in CVJ, a recent report of atlantoaxial pneumoarthrosis due to hyperpneumatized temporal bone showed surgical closure of communication with bone and fat grafts, resulting in relieving symptoms of neck pain and tinnitus [19].

USG has been used both clinically and experimentally, and it showed the detection of echogenic foci due to intra-articular air even in the presence of concomitant fluid. USG can detect a smaller quantity of air (0.5 ml), whereas x-rays require at least 2 ml of air. CT has been considered the gold standard as the Hounsfield values of air are distinct and obvious. MRI has been shown to mislead about the presence of intra-articular air, but a recent study using 3T MRI has shown that gradient recalled echo (GRE) and 3D sequences can delineate the presence of intra-articular air [2,6,8,9].

## Conclusions

Pneumoarthrosis or air inside a joint may be a benign condition. It may be seen following typical positions of the limb and may be more noticeable with traction. However, the patient must be evaluated with proper history and imaging, such as radiograph and CT scan, if it persists or there is suspicion of a disease pathology. Most of the time history of trauma and degeneration are associated with the pneumoarthrosis.
